# Salt Stress Enhances Aroma Component 2-Acetyl-1-pyrroline in Aromatic Coconut (*Cocos nucifera* Linn.)

**DOI:** 10.3390/plants15020174

**Published:** 2026-01-06

**Authors:** Jinyao Yin, Dan Luo, Cuinan Shi, Hao Ding, Jing Li, Xiwei Sun, Xiaojun Shen, Xiaomei Liu, Amjad Iqbal, Yaodong Yang

**Affiliations:** 1 National Key Laboratory of Tropical Crop Breeding/Hainan Key Laboratory of Tropical Oil Crops Biology, Coconut Research Institute, Chinese Academy of Tropical Agricultural Sciences, Wenchang 571339, China; yin0728@catas.cn (J.Y.); amjadiqbal@awkum.edu.pk (A.I.); 2 Hainan Coconut International Joint Research Center, Wenchang 571339, China; 3 Department of Food Science & Technology, Abdul Wali Khan University Mardan, Mardan 23200, Pakistan

**Keywords:** aromatic coconut, 2-acetyl-1-pyrroline, salt stress, antioxidant enzyme activity, biosynthesis mechanism

## Abstract

Aromatic coconut (*Cocos nucifera* L.) is valued in the consumer market primarily for its distinctive fragrance, which is largely attributed to the compound 2-acetyl-1-pyrroline (2AP). The accumulation of 2AP has been observed in several crops, such as rice, when exposed to salt stress. In rice, exposure to salt stress influences the activity of enzymes, alters amino acid metabolism, and modulates the expression of genes associated with 2AP formation. Nevertheless, the processes responsible for 2AP biosynthesis in aromatic coconut under salt stress conditions are still not well clarified. In this study, five-month-old aromatic coconut seedlings were subjected to four distinct levels of sodium chloride (NaCl) treatment (0, 100, 200, and 300 mM). This experiment was conducted to investigate the mechanisms involved in salt-induced responses and the biosynthesis of 2AP in aromatic coconut. Although salt stress did not produce any apparent injury in the coconut seedlings, it led to a marked decline in chlorophyll content. Meanwhile, salt stress markedly enhanced the accumulation of betaine and boosted the activities of antioxidant enzymes such as superoxide dismutase and catalase. The aromatic coconut demonstrated a moderate level of salt tolerance. Salt stress also had a significant influence on 2AP biosynthesis. Under salt stress conditions, the 2AP content increased substantially, reaching its highest level with a 93.55% rise compared to the control. Furthermore, the synthesis of 2AP in aromatic coconut under salt stress appears to be primarily regulated through the metabolic pathways of proline and glutamate. Therefore, salt stress enhances 2AP production, with 200 mM NaCl identified as the optimal concentration for its accumulation.

## 1. Introduction

Coconut (*Cocos nucifera* L.) is a perennial evergreen tree belonging to the genus *Cocos* of the family Arecaceae, comprising both tall and dwarf varieties. Among them, the aromatic coconut, a distinctive type of green dwarf coconut, is well known for its characteristic pandan-like fragrance [1,2]. Aroma compounds are not only crucial determinants of fruit flavor quality, but also play a vital role in influencing consumer preference and purchase behavior [3,4]. The characteristic fragrance of aromatic coconut resembles that of *Oryza sativa* L., *Haraella retrocalla* (Hayata) Kudô, and *Vallaris glabra* [5]. Of these compounds, 2-acetyl-1-pyrroline (2AP), originally discovered in cooked rice, has been recognized as the key molecule contributing to the characteristic aroma [6].

To date, amino acids, including ornithine, glutamate, and proline, have been recognized as major precursors involved in the biosynthetic pathway of 2AP [7,8]. External supplementation of proline has been reported to elevate the 2AP concentration in aromatic rice varieties [9]. Similarly, hydroponic experiments using L-glutamate in fragrant rice demonstrated enhanced levels of 2AP and proline, along with increased activity of pyrroline-5-carboxylate synthetase (P5CS) [7,10]. Additionally, treatment with exogenous γ-aminobutyric acid (GABA) has been reported to promote 2AP accumulation and increase the yield of aromatic rice when exposed to low-light environments [11]. The enhanced production of 2AP in rice grains has been linked to increased levels of proline and pyrroline-5-carboxylic acid (P5C), along with elevated activities of key enzymes, such as ornithine aminotransferase (OAT), proline dehydrogenase (PRODH) and P5CS [12]. Moreover, studies on rice have revealed that the biosynthesis of 2AP primarily occurs through two main metabolic pathways [13,14,15]. One pathway involves the precursors proline and glutamate, which are enzymatically converted into P5C. Through a series of dicarboxylic reactions, P5C is subsequently transformed into Δ^1^-pyrroline, which then participates in the final synthesis of 2AP [16,17]. The second pathway involves a non-enzymatic reaction between the key metabolite methylglyoxal and other intermediates, leading to the formation of the aromatic compound 2AP [18]. In soybeans, it was noticed that the genes associated with 2AP biosynthesis, such as pyrroline-5-carboxylate reductase (P5CR), PRODH, P5CS and pyrroline-5-carboxylate dehydrogenase (P5CDH), are highly expressed in the aromatic soybean cultivar ZK1754 [19]. The compound 2AP has been detected in the endosperm of aromatic coconut varieties but is absent in non-aromatic types [20]. A previous study identified amino aldehyde dehydrogenase 2 (*CnAMADH2*) as an ortholog of the rice aroma-related gene [20]. Moreover, a G-to-C substitution in exon 14 of this gene correlates with 2AP accumulation in aromatic coconuts. In addition, previous genome-wide association studies have reported 32 loci that are significantly linked to 2AP content in aromatic coconut varieties [21]. Despite these findings, the molecular mechanism underlying 2AP biosynthesis in aromatic coconuts remains largely unclear.

Aromatic products are considered high-value commodities and often command premium market prices [2]. Therefore, enhancing aroma production has become an important objective and challenge in related research and industrial fields. Previous studies have shown that salt treatment can stimulate 2AP biosynthesis, thereby enhancing aroma formation in fragrant rice leaves [22,23]. When exposed to salt stress, rice leaves exhibited 2AP levels between 733 and 998 μg·kg^−1^, significantly exceeding the 592 μg·kg^−1^ observed in the control group. Furthermore, exposure to salt stress caused both proline and 2AP levels in the leaves to peak [22]. Research on two aromatic rice cultivars, Basmati-370 and Ambemohar-157, indicated that ornithine, proline, and methylglyoxal together play a key role in enhancing 2AP synthesis under saline conditions [17].

Salt stress, a predominant abiotic stressor, stimulates the excessive formation of reactive oxygen species (ROS), which subsequently compromises the integrity of plant cellular membranes [24,25]. In response, plants activate antioxidant defense mechanisms, resulting in altered activities of key enzymes, such as peroxidase (POD), superoxide dismutase (SOD), and catalase (CAT) to scavenge ROS and mitigate oxidative damage [26,27,28,29]. Additionally, earlier research has reported the identification of multiple genes involved in the salt stress response in coconut. The coding sequence of Cu-Zn superoxide dismutase (SODCP) was found to be upregulated in Hainan tall coconut, contributing to its enhanced salt tolerance [30]. In contrast, dwarf coconut varieties may have lost the SODCP gene during the course of human domestication [31]. Additionally, a protein phosphatase 2C gene involved in the signaling cascade mediated by abscisic acid (ABA) was downregulated in coconut under salt stress, whereas it is typically upregulated in rice, *Arabidopsis*, and other model plants [30,32]. The mechanism underlying coconut’s response to salt stress remains a key research focus and a major challenge, as many aspects are still poorly understood and require further investigation. To better understand the adaptive mechanisms of aromatic coconut under saline conditions, seedlings were exposed to varying levels of NaCl stress (100, 200, and 300 mM). The study assessed chlorophyll and betaine accumulation, activities of the antioxidant enzymes CAT and SOD, and the concentrations of P5C. In addition, the enzymatic functions associated with the biosynthetic pathway of 2AP, i.e., P5CS, ODC, DAO, and GABA, were also analyzed. We also primarily analyzed the 2AP biosynthesis mechanism in aromatic coconut under salt stress, revealing that it is induced through the promotion of metabolic cycling of proline and glutamate.

## 2. Results

### 2.1. Aromatic Coconut Is a Moderately Salt-Tolerant Plant

**Figure 1:**
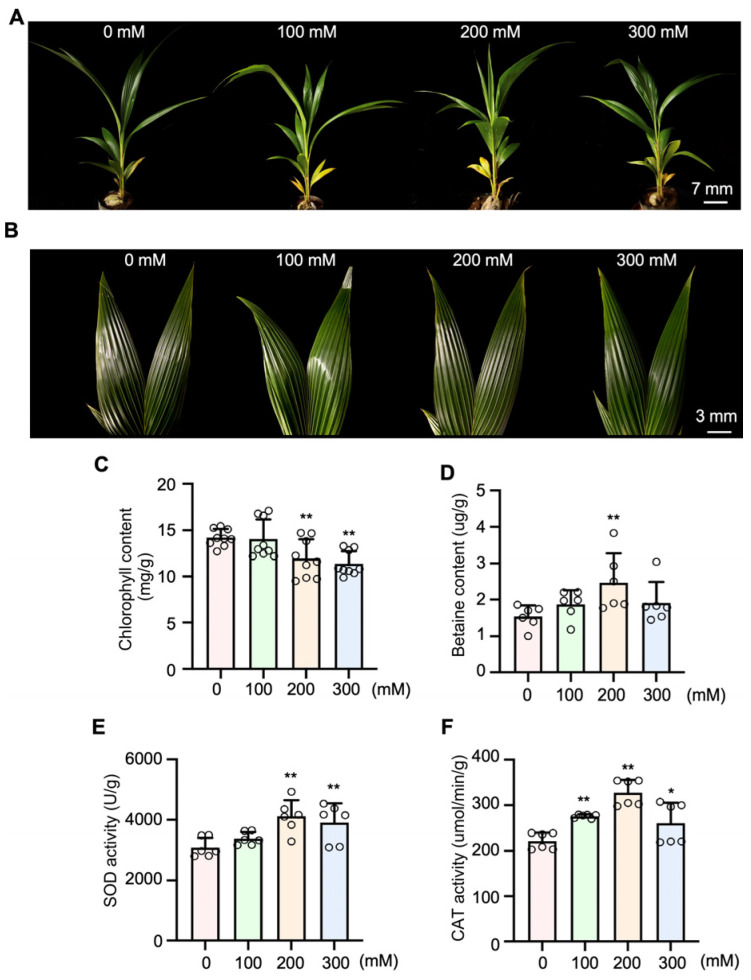
The response of aromatic coconuts to salt stress. The growth of aromatic coconut (**A**) seedlings and (**B**) leaves under salt stress. In (**A**,**B**), the aromatic coconuts were exposed to 0, 100, 200 and 300 mM NaCl for 6 days and then photographed. The contents of (**C**) chlorophyll and (**D**) betaine in aromatic coconut leaves treated with different NaCl concentrations. The enzyme activities of (**E**) SOD and (**F**) CAT in aromatic coconut leaves were measured. In (**C**–**F**), data points (*n* = 9 in (**C**); *n* = 6 in (**D**,**E**)) are from three independent experiments. Error bars represent standard deviation (SD), and asterisks represent significant differences compared with the control (0 mM NaCl treatments), as analyzed by Student’s *t*-tests (*, *p* ≤ 0.05; **, *p* ≤ 0.01).

In order to assess the influence of salt stress on aromatic coconut, seedlings were exposed to varying NaCl concentrations of 0, 100, 200, and 300 mM. Throughout the treatment period, leaf etiolation gradually appeared six days after treatment (Figure 1A,B), which suggests that aromatic coconut exhibits a certain degree of salt tolerance. Relative to the control group (0 mM NaCl), salt stress led to a significant decline in chlorophyll content and a pronounced rise in betaine accumulation after 3 days of treatment. The greatest decrease in chlorophyll content (19.72%) was observed at 300 mM NaCl treatment for 3 days, whereas the highest increase in betaine content (62.68%) occurred at 200 mM NaCl (Figure 1C,D). Likewise, the activities of the antioxidant enzymes SOD and CAT were examined to evaluate the occurrence of oxidative stress under high salinity conditions. Both enzyme activities increased notably under the 200 mM NaCl treatment for 3 days (Figure 1E,F).

### 2.2. Expression Profiles of Differentially Expressed Genes Under Varied Salt Stress

**Figure 2:**
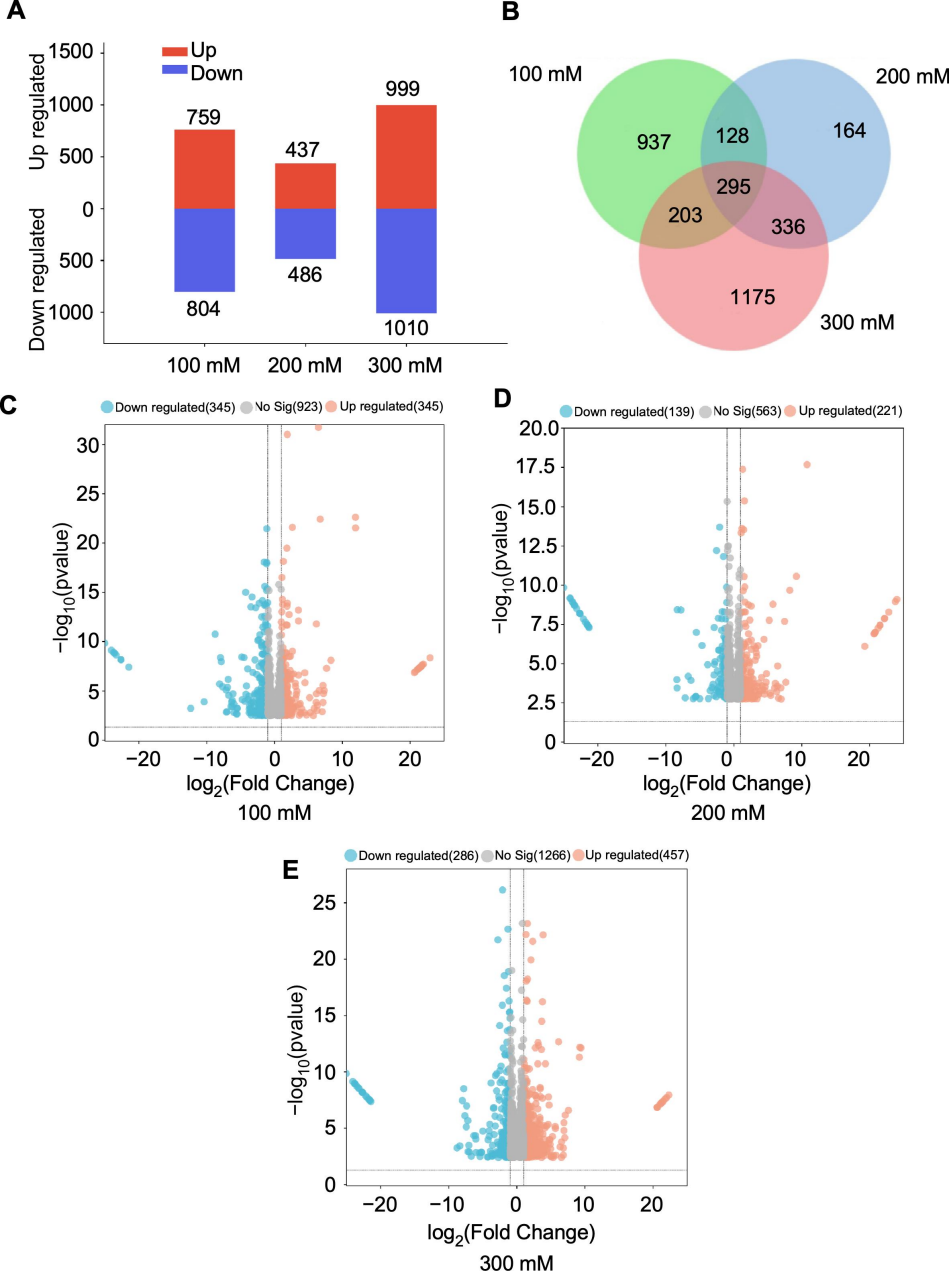
DEGs in aromatic coconuts under salt stress. (**A**) The maps of DEGs in coconut leaves under 100, 200, and 300 mM NaCl treatments, compared with 0 mM NaCl treatment (control). (**B**) Venn diagram of differential gene co-expression between groups after 100, 200, and 300 mM NaCl stress. Volcano visualization of DEGs in (**C**) 0 vs. 100 mM NaCl, (**D**) 0 vs. 200 mM NaCl, and (**E**) 0 vs. 300 mM NaCl comparisons.

RNA sequencing was performed to detect differentially expressed genes (DEGs) by comparing control seedlings (0 mM NaCl) with those subjected to 100, 200, and 300 mM NaCl treatments. A total of 1563, 923, and 2009 DEGs [false discovery rate (FDR) < 0.05; log2 fold change (|LFC|) > 0.5] were identified in the 100, 200, and 300 mM NaCl treatments for 3 days, relative to the control (Figure 2).

**Figure 3:**
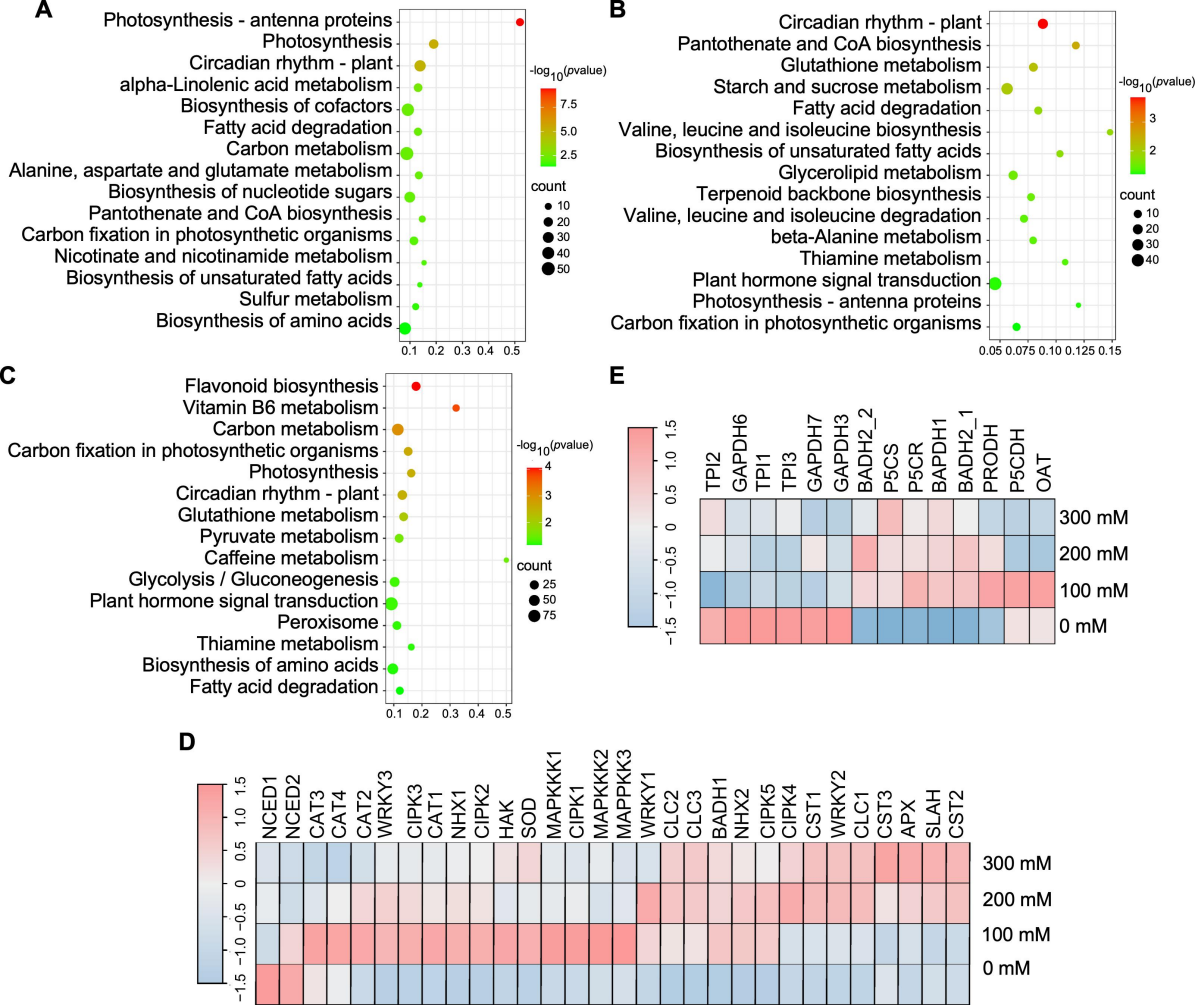
The transcriptome analysis of aromatic coconut leaves’ response to salt stress. Enrichment analysis of KEGG pathways of aromatic coconut under (**A**) 100 mM NaCl, (**B**) 200 mM NaCl, and (**C**) 300 mM NaCl treatments. The 15 pathways in which *p* value ≤ 0.05 are primarily shown. (**D**) Heat map of DEGs related to salt tolerance. (**E**) Heat map of DEGs in the 2-acetyl-1-pyrroline (2AP) synthesis pathway. In (**D**,**E**), the DEGs were filtered based on significance (FDR < 0.05) and expression level (LFC > |0.5|). The count numbers for each sample were normalized using the DESeq2 package in R (version 1.38.3). The plotted data are homogenized averages from 3 repeat experiments.

Gene Ontology (GO) classification of these DEGs revealed variations among treatments, with 36, 31, and 33 functional categories identified under 100, 200, and 300 mM NaCl treatments, respectively (Figure S1). KEGG pathway annotation revealed that under 100, 200, and 300 mM NaCl treatments, most pathways in aromatic coconut were classified under the “Metabolism” category, accounting for 76.4%, 76.9%, and 77.6% of the total pathways, respectively (Figure S2). KEGG enrichment analysis showed that Photosynthesis–antenna proteins and photosynthesis pathways were significantly enriched under 100 mM NaCl treatment (Figure 3A), whereas starch and sucrose metabolism and carbon metabolism pathways were remarkably enriched under 200 mM and 300 mM NaCl treatments (Figure 3B,C).

### 2.3. Salinity Triggers Salt-Tolerant Gene and 2AP-Synthesis-Related Gene Expression

**Figure 4:**
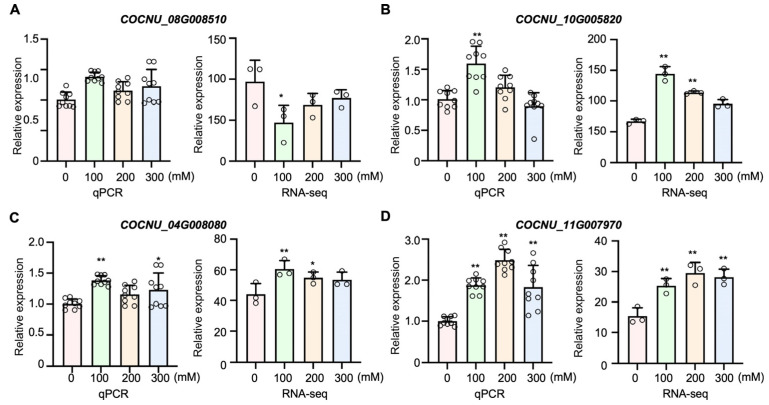
The expression levels of DEGs in aromatic coconuts under salt stress. (**A**) *CnTPI* (COCNU_08G008510); (**B**) *CnCIPK* (COCNU_10G005820); (**C**) *CnP5CR* (COCNU_04G008080); (**D**) *CnCLC* (COCNU_11G007970). qPCR, reverse transcription-quantitative PCR (RT-qPCR) analysis; RNA-seq, RNA transcriptome sequencing data analysis. Data points (*n* = 6 for RT-qPCR; *n* = 3 for RNA-seq) are from three independent experiments. Error bars represent SD, and asterisks represent significant differences compared with the control (0 mM NaCl treatments), as analyzed by Student’s *t*-tests (*, *p* ≤ 0.05; **, *p* ≤ 0.01).

We applied a logarithmic function with base 10 [Log_10_ FRKM ≥ |0.5|] to screen for salt-tolerant genes, resulting in the identification of 31 DEGs. Among these, the transcript level of epoxy-type carotenoid dioxygenase genes (*CnNCED1* and *CnNCED2*) was downregulated to varying degrees across all three salt concentration treatments. The betaine aldehyde dehydrogenase gene (*CnBADH1*), the chloride channel protein genes (*CnCLC2* and *CnCLC3*), and the Na^+^/H^+^ transporter genes (*CnNHX1* and *CnNHX2*) were upregulated in response to the three different salt stress concentrations. Interestingly, the expression patterns of other salt-tolerance-related genes varied depending on the level of salt stress. The calcineurin B-like interacting protein kinase genes, *CnCIPK4* and *CnCIPK2*, were upregulated when treated with 100, 200, and 300 mM NaCl compared to the control. However, *CnCIPK5* was upregulated only under high salinity conditions (200 and 300 mM NaCl) (Figure 3D). In addition, 13 DEGs associated with the synthesis of 2AP were identified. Remarkably, *CnP5CS* and *CnP5CR*, which encode enzymes involved in the conversion of P5C, were significantly upregulated under all three salt concentration treatments (Figure 3E). In contrast, the transcript levels of the triosephosphate isomerase genes (*CnTPI1*, *CnTPI2*, and *CnTPI3*) and the glyceraldehyde-3-phosphate dehydrogenase genes (*CnGAPDH3*, *CnGAPDH6*, and *CnGAPDH7*) were markedly reduced (Figure 3E). The qRT-PCR analysis of four randomly selected genes related to 2AP biosynthesis and salt tolerance exhibited expression trends consistent with the RNA-seq data (Figure 4).

### 2.4. High Salinity Induces the Synthesis of 2AP

**Figure 5:**
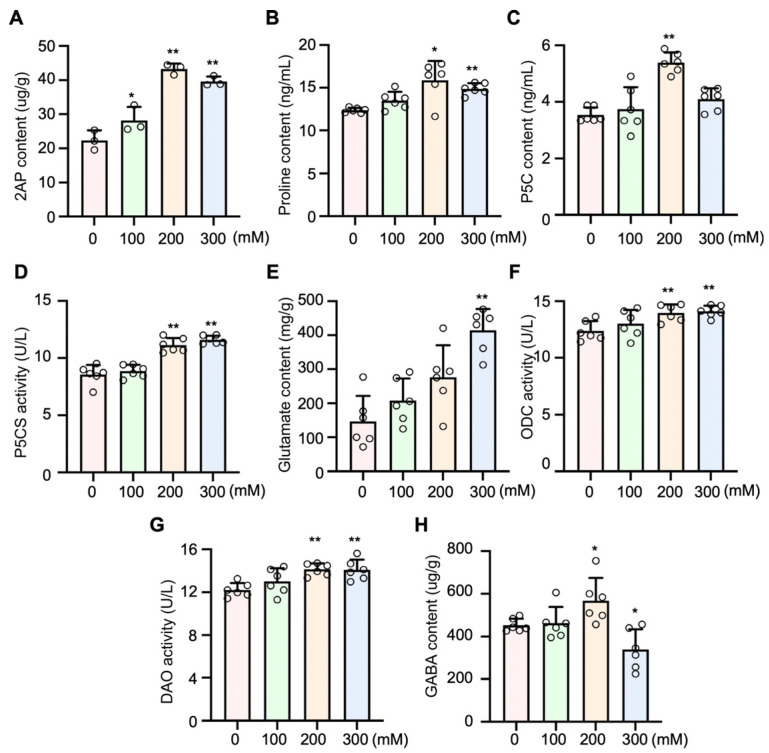
Salt stress influences the physiological indexes related to the synthesis of 2AP in aromatic coconuts. (**A**) The 2AP content in coconut leaves increased under salt stress, reaching its highest level under 200 mM treatments. Data points (*n* = 3) are from three independent experiments. The variation in (**B**) proline and (**C**) P5C contents under NaCl treatments has a positive correlation with 2AP contents, with the maximum observed under the 200 mM treatment. The (**D**) P5CS activity and (**E**) glutamate content in aromatic coconut increased with increasing NaCl concentrations, reaching their highest levels under the 300 mM treatment. The activity of (**F**) ODC and (**G**) DAO also showed a positive correlation with 2AP contents, while (**H**) GABA exhibited a negative correlation, with their extreme levels observed under 200 mM NaCl treatments. In (**B**–**H**), data points (*n* = 6) are from three independent experiments. Error bars represent SD and asterisks represent the significant differences compared with the control (0 mM NaCl treatments), as analyzed by Student’s *t*-tests (*, *p* ≤ 0.05; **, *p* ≤ 0.01).

To explore the effects of salinity on 2AP production, we determined its concentration in coconut seedlings exposed to multiple salt treatments for 3 days. The results showed that 2AP levels increased by 26.1%, 93.6%, and 77.1% under 100, 200, and 300 mM NaCl treatments, respectively (Figure 5A and Figure S3). In addition, the levels of proline and P5C increased by 28.46% and 52.41%, respectively, under 200 mM NaCl treatment (Figure 5B,C). The P5CS activity increased significantly under 200 mM and 300 mM salinity, with the highest level observed at 300 mM (Figure 5D). Interestingly, the content of glutamate showed significant increases when treated with 300 mM NaCl (Figure 5E). Meanwhile, the activities of ODC and DAO were significantly enhanced under 200 and 300 mM NaCl treatments (Figure 5F,G). Furthermore, the GABA content increased when treated with 200 mM NaCl, whereas it decreased when treated with 300 mM NaCl (Figure 5H).

## 3. Discussion

The aromatic coconut is well known for its distinctive pandan-like fragrance that emanates from its leaves, water, and meat [2,20]. However, this characteristic aroma is unstable and can be affected by environmental conditions, temperature, and various other factors [20]. Consequently, enhancing the concentration of 2AP, the primary compound responsible for the aroma, has become a major challenge limiting the further development of the aromatic coconut industry. Previous studies on aromatic rice have reported that NaCl treatment enhances the accumulation of 2AP [17]. Similarly, our results showed that 2AP content in coconut seedlings increased by 93.55% under 200 mM NaCl treatment compared with the 0 mM NaCl control group. These results indicate that NaCl treatment can induce an increase in 2AP content in coconuts.

Moreover, past studies have indicated that coconuts are a moderately salt-tolerant glycophyte. However, research has shown that coconut seedlings experience severe damage when exposed to seawater concentrations as high as 50% for a duration of six months [33,34]. Our study revealed that exposure to high salinity levels (100, 200, and 300 mM NaCl) for 6 days had an impact on the appearance of young aromatic coconut seedlings, with mild chlorosis observed in newly emerged leaves. These observations suggest that aromatic coconut seedlings possess a certain degree of salt tolerance.

However, chlorophyll content declined progressively with increasing NaCl concentration, indicating that salt stress adversely affects the photosynthetic efficiency of the seedlings. In addition, our results showed that betaine content and the activities of CAT and SOD initially increased under salt stress and then declined at higher salinity levels. Similar patterns have been reported in cotton, where SOD and CAT activities were found to increase progressively with elevated salt levels (0–150 mM NaCl) [35]. Interestingly, in pepper, the activities of CAT and SOD exhibited a different trend, first decreasing and then increasing under mixed salt concentrations ranging from 0 to 20 g/L [36]. We speculate that such variations may result from species-specific differences in enzymatic responses to varying salt levels. Furthermore, our analysis showed that the transcript level of *CnSOD* was upregulated in coconut seedlings treated with three concentrations of NaCl, with the strongest induction observed at 100 mM. These results suggest that in aromatic coconut, SOD may function as the primary line of defense against oxidative stress induced by salinity, leading to enhanced enzyme activity under moderate salt conditions. As the salt concentration increases, the levels of ROS rise, leading to enhanced activity of antioxidant enzymes, such as CAT, which help mitigate the effects of high salinity stress. This trend continues until the plants gradually adapt to the salt-stressed environment, after which enzyme activity levels decline and approach their original baseline. In general, aromatic coconut seedlings appear to mitigate salt-induced stress by regulating osmotic balance and maintaining ROS homeostasis.

To further investigate the molecular mechanisms underlying salt stress tolerance in coconut, we conducted a comparative transcriptomic analysis of seedlings treated with 100, 200, and 300 mM NaCl for 3 days, using 0 mM NaCl as the control. It is well established that salt stress induces osmotic imbalance and water deficit in plants, which can severely disrupt cellular homeostasis and metabolic processes [37,38]. Under normal conditions, osmotic signaling triggers the accumulation of endogenous ABA, which promotes stomatal closure to maintain water status and osmotic balance [39]. Nine-cis-epoxycarotenoid dioxygenases (*NCEDs*) serve as key rate-limiting enzymes in the ABA biosynthetic pathway, playing a crucial role in regulating ABA production in plants [40,41]. In *Arabidopsis thaliana*, upregulation of *NCED3*, a critical enzyme in ABA synthesis, enhances ABA accumulation and contributes to improved tolerance against salt stress [42]. Similarly, in barley, *HvNCED1* expression is markedly upregulated under salt stress, whereas *HvNCED3* and *HvNCED4* showed downregulation, indicating species- and isoform-specific responses to salinity [41]. In our study, the transcription levels of *CnNCED1* and *CnNCED2* genes were downregulated under salt stress, suggesting that the regulatory mechanism of ABA biosynthesis in response to salinity is more complex than previously understood. Noticeably, both aromatic coconut and barley exhibit relatively high salt tolerance, which contrasts with the more salt-sensitive response observed in *A. thaliana*. This suggests that their salt stress responses and associated hormonal signaling pathways are more intricate. Previous studies have reported that WRKY transcription factors are functionally linked to the MAPK signaling cascade, which plays a pivotal role in mediating plant responses to salt stress [43]. We observed that the expression levels of *CnWRKY1* and *CnWRKY2* were upregulated under 200 mM NaCl treatment. The transcript levels of *CnMAPKK1*, *CnMAPKK2*, and *CnMAPKK3* were all downregulated under 200 mM NaCl treatment. Based on this, we hypothesize that a potential interaction may exist between *CnWRKY* and *CnMAPKKK*. In our future work, we aim to further investigate this hypothesis.

Moreover, the levels of proline, glutamate, and P5C, as well as the activity of P5CS, showed a positive correlation with 2AP accumulation. Interestingly, the contents of proline and P5C increased significantly, reaching their highest levels under the 200 mM NaCl treatment. In aromatic rice, proline has been identified as a key precursor in 2AP biosynthesis, and elevated proline levels promote its conversion into 2AP [44]. Similarly, in soybean, P5C, derived from L-proline, L-glutamic acid, or L-ornithine, can react directly with methylglyoxal to form 2AP [45]. Previous studies have also reported that upregulation of P5C, Δ^1^-pyrroline, and proline, along with the downregulation of glutamic acid under optimal trans-zeatin concentrations, enhanced 2AP biosynthesis in fragrant rice seedlings [46]. This suggests that the increased P5C content observed in aromatic coconuts under salt stress may be a key factor contributing to the elevated 2AP levels.

**Figure 6:**
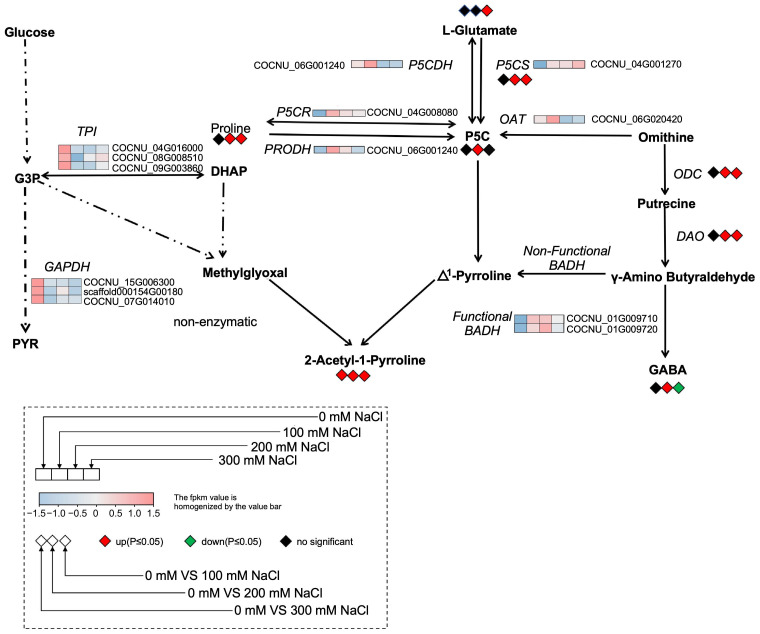
The biosynthetic pathway of 2AP in aromatic coconut under salt stress. P5CS, Δ^1^-Pyrroline-5-carboxylic acid synthetase; P5CR, Pyrroline-5-carboxylate reductase; PRODH, Proline dehydrogenase; TPI, Triosephosphate isomerase; GAPDH, Glyceraldehyde-3-phosphate dehydrogenase; OAT, Ornithine aminotransferase; ODC, Ornithine decarboxylase; DAO, Diamine oxidase; P5C, Δ^1^-Pyrroline-5-carboxylic acid; BADH, Betaine aldehyde dehydrogenase; GABA, γ-Aminobutyric acid. Red, green and black indicate upregulated, downregulated, and no significant change, respectively. The rectangle represents the regulated levels of DEGs in coconuts under different NaCl treatments, while the rhombus represents the variation trends of target compounds in coconuts under different NaCl treatments, relative to the control (0 mM NaCl treatment).

To further investigate the mechanism by which salt stress increases P5C content, we analyzed the expression of genes involved in P5C biosynthesis. Compared with the control, the expression levels of *CnP5CR* and *CnPRODH* were upregulated when treated with 200 mM NaCl. Similarly, the contents of P5C and proline also reached their highest levels under the 200 mM NaCl treatment. These findings indicate that, under salt stress, P5C in coconuts may be primarily synthesized via the proline pathway (Figure 6). Therefore, we propose that glutamate is likely the primary resource for synthesizing P5C in aromatic coconut under the 200 mM treatment. Previous studies have also reported that upregulation of P5CS correlates with enhanced 2AP accumulation in aromatic soybean [45]. In addition, we observed that under the 300 mM NaCl treatment, P5CS enzyme activity and glutamate content reached their highest levels. Similar trends have also been observed in tea and sesame, where salt stress promotes glutamate accumulation [47,48]. Meanwhile, we found that the expression of *CnP5CS* was not only upregulated by NaCl treatment but also reached its maximum level at 300 mM. These findings partially support our hypothesis that both proline and glutamate pathways serve as the primary routes for P5C synthesis in coconuts (Figure 6). The exact relationship between proline, glutamate, P5C, and 2AP biosynthesis needs to be explored and demonstrated in our future work.

Past studies have reported that under salt stress and shading conditions, the contents of 2AP and GABA in the grains of the rice cultivar ‘Yuxiangyouzhan’ both increases, exhibiting a significant positive correlation between the two compounds [22,49]. In our study, we observed that GABA content increased under 200 mM NaCl treatment but was downregulated under 300 mM NaCl treatment. The activity of *CnBADH2*, which catalyzes the conversion of GABald to GABA, exhibited a trend similar to that of GABA content. The increased GABald may promote the formation of Δ^1^-pyrroline in plants with non-functional BADH under high salinity conditions, thereby further enhancing 2AP synthesis. However, the precise mechanism of 2AP biosynthesis remains unclear and requires further investigation, particularly in coconuts.

## 4. Materials and Methods

### 4.1. Plants Culture and Salt Stress Treatment

The coconut seedlings used in this study were from the “Wenye No. 4” cultivar, an aromatic coconut (*Cocos nucifera* L.) variety suitable for cultivation in China. The seedlings were cultivated in the coconut plantation of the Coconut Research Institute, Chinese Academy of Tropical Agricultural Sciences (110.80° E, 19.56° N) in Wenchang, Hainan, China. We transplanted these seedlings after 5 months into plastic pots (39 cm in upper diameter, 23 cm in lower diameter, and 29 cm in height). The pots were filled with a mixture of neutral pH soil and coconut bran (1:1, *v*/*v*) and cultivated in a plant growth chamber under controlled conditions (16 h light/8 h dark photoperiod at 23 °C). After a seven-day acclimation period, the roots were irrigated daily with 300 mL of NaCl solutions (0 mM, 100 mM, 200 mM, and 300 mM) prepared in double-distilled water. After six days of NaCl treatment, the seedlings were photographed. After three days of treatment, leaf samples were collected from identical positions across all seedlings for further analyses.

### 4.2. RNA Extraction and RNA-Seq Analysis

For RNA extraction, aromatic coconut leaf samples were ground in liquid nitrogen, and total RNA was isolated using the Spin Column Plant Total RNA Purification Kit (B518661, Sangon Biotech, Shanghai, China). The quality of the extracted RNA was first assessed by 1% agarose gel electrophoresis, while RNA integrity and concentration were determined using an Agilent 2100 Bioanalyzer (Agilent Technologies, Santa Clara, CA, USA) and a NanoDrop spectrophotometer (IMPLEN, Westlake Village, CA, USA), respectively. Subsequently, RNA sequencing was carried out on the BGISEQ-MGI2000 platform at BGI Genomics (Wuhan, China). Following sequencing, raw reads were processed to obtain clean data by removing adapter-contaminated reads, reads with >5% unknown bases, and low-quality reads (where >20% of bases had a quality score ≤ 10). The resulting clean reads were stored in FASTQ format and aligned to the reference genome with HISAT2 (version 2.1.0), followed by mapping to the assembled unique genes using Bowtie2 (version 2.2.5). Gene expression levels were quantified with RSEM (version 1.2.8). Unigenes were functionally annotated against KEGG and GO databases. Differential gene expression analysis was conducted with DESeq2 under the thresholds of |Fold Change| ≥ 2 and adjusted *p*-value ≤ 0.001.

### 4.3. qRT-PCR to Analyze Target Gene Transcript Levels

Complementary DNA (cDNA) was synthesized from total RNA (0.001 g/µL, 1 μL) using the PrimeScript™ II 1st Strand cDNA Synthesis Kit (6210, TaKaRa, Shiga, Japan). Quantitative real-time PCR (qRT-PCR) was then conducted on a QuantStudio™ 6 Flex system (Applied Biosystems, Foster City, CA, USA) using the PowerUp^TM^ SYBR^TM^ Green Master Mix (Thermo Fisher Scientific, Waltham, MA, USA). The *CnACT* gene (GenBank accession: GABT01020912.1) served as the internal reference for normalizing gene expression levels. Primer sequences are listed in Table S1. The PCR amplification was performed under the following thermal cycling conditions: 95 °C for 5 s, followed by 40 cycles of 95 °C for 5 s, 58 °C for 30 s, and 72 °C for 30 s.

### 4.4. Determination of 2AP Contents in Leaves

The 2AP content in coconut leaves was quantified using gas chromatography-mass spectrometry (GC-MS), following the method described by Jirapong et al. [50]. Briefly, 1 g of leaf powder from each sample was ground in liquid nitrogen and extracted with 2 mL of absolute methanol. At the same time, 10 µL of 2 ng/µL 2,4,6-trimethylpyridine (TMP, 99% purity) was added to the bottle as an internal standard. The mixture was subjected to ultrasonic treatment at 80 °C for 240 min, after which the supernatant was filtered through a 0.22 μm syringe filter. Subsequently, 1 mL of the filtrate was injected into the GC-MS system for analysis. The gas chromatography system (7890B, Agilent Technologies, Santa Clara, CA, USA) was utilized to analyze the content of 2AP in the samples. The high-purity helium (He > 99.999%) was used as the carrier gas. The injection volume was 1 mL with a solvent delay of 3 min, and the flow rate was maintained at 1 mL/min. The temperature program was set as follows: hold at 45 °C for 1 min, increase to 100 °C at a rate of 5 °C/min, then ramp to 280 °C at 300 °C/min. The electron ionization (EI) mode of mass spectrometry (MS) was employed with an electron energy of 70 eV. The ion source temperature was set to 230 °C, and the scanning range was *m*/*z* 20–280. The 2AP concentration in leaf tissues was determined based on a calibration curve (Y = 204.6x + 208.1; R^2^ = 0.9957).

For the determination of P5C content in coconut leaves, 0.1 g of leaf tissue was homogenized with 1 mL of 3% sulfosalicylic acid. The homogenate was centrifuged, and the resulting supernatant was mixed with 10% trichloroacetic acid and 2-aminoanisaldehyde. After incubation for 30 min, the mixture was centrifuged again, and the supernatant was transferred to a 96-well microplate. The absorbance was recorded at 440 nm using an LB941 microplate reader (Berthold Technologies, Bad Wildbad, Germany) to quantify P5C content.

### 4.5. Measurement of Physiological Indicators

For chlorophyll determination, 0.1 g of fresh leaf tissue was homogenized in 1 mL of Chlorophyll Assay Buffer (R30354-50T, Orileaf, Shanghai, China). The absorbance of the extract was measured at 665 nm and 649 nm using a spectrophotometer (Mapada UV-1800PC, Shanghai, China), and chlorophyll content was calculated accordingly. For betaine analysis, 0.1 g of fresh leaf tissue was extracted with 1 mL of 80% methanol, and the homogenate was centrifuged at 12,000 rpm for 15 min in a centrifuge (Centrifuge 5804R, Eppendorf, Hamburg, Germany). The absorbance of the resulting supernatant was then measured at 340 nm using a microplate reader (Infinite E Plex, Tecan, Männedorf, Switzerland) following the manufacturer’s instructions (G0122W, Grace, Suzhou, China).

Leaf samples were ground and homogenized with the specific extraction buffer (1 mL per 0.1 g of sample) for glutamate, SOD, and CAT, respectively. The mixture was then centrifuged at 12,000 rpm and 4 °C in a centrifuge (Centrifuge 5804R, Eppendorf, Hamburg, Germany) for 10 min. The supernatant was collected and kept on ice for subsequent measurement. The absorbance of the supernatant was measured at 450 nm using a microplate reader (Infinite E Plex, Tecan, Männedorf, Switzerland). The values were calculated using the plant MDA content determination kit (G0416W, Grace, Suzhou, China) and the plant SOD assay kit (G0102W, Grace, Suzhou, China). Similarly, CAT activity was quantified using the plant CAT assay kit (G0105W, Grace, Suzhou, China) by measuring the absorbance of the supernatant at 510 nm with the same instrument.

Leaf samples (0.1 g) were ground into a fine powder and homogenized with 900 μL of 1× phosphate-buffered saline (PBS) solution. The homogenate was centrifuged at 12,000 rpm and 4 °C for 10 min using a centrifuge (Centrifuge 5804R, Eppendorf, Hamburg, Germany). The resulting supernatant was used to determine proline levels and the activities of P5CS, ODC, and DAO according to the respective kit protocols. Proline content was measured using the plant proline content determination kit (G0111W, Grace, Suzhou, China) by assessing the absorbance of the supernatant at 520 nm with a microplate reader (Infinite E Plex, Tecan, Männedorf, Switzerland). The activities of P5CS, ODC, and DAO were determined using the plant P5CS ELISA kit (MB-10521A, Mbbiology, Yanchengan, China), the plant ODC ELISA kit (MB-4792A, Mbbiology, Yancheng, China), and the plant DAO ELISA kit (MB-5091A, Mbbiology, Yancheng, China), respectively, by measuring the absorbance of the supernatant at 450 nm with the same instrument.

### 4.6. Statistical Analysis

The Shapiro–Wilk test was used to assess the normality of data, and Student’s *t*-test was used to compare two groups, with *p* < 0.05 or *p* < 0.01 considered statistically significant. Heatmaps of differentially expressed genes (DEGs) were generated using TBtools II (Version 2.330) [51]. SPSS software (version 20.0, SPSS, Chicago, IL, USA) was utilized for data analysis. Tables and graphs were generated using Graphpad Prism (version 9.5, GraphPad Software, San Diego, CA, USA). Correlation coefficients were calculated based on the mean values.

## 5. Conclusions

This study demonstrates that aromatic coconut possesses a moderate level of salt tolerance, as evidenced by the activation of antioxidant defenses despite a reduction in chlorophyll content. Exposure to salt stress markedly enhanced betaine accumulation and increased the activities of antioxidant enzymes, SOD and CAT, which likely contributed to mitigating oxidative damage. Moreover, salinity stress significantly promoted the accumulation of 2AP, with the highest levels observed under 200 mM NaCl treatment. The precursors of 2AP accumulation, including proline, P5C, and glutamate, exhibited variable increases in response to different NaCl concentrations. Collectively, these findings indicate that moderate salt stress not only triggers adaptive physiological responses but also enhances 2AP accumulation in aromatic coconut, providing a potential strategy to improve aroma quality through controlled salinity management.

## Data Availability

The datasets generated and/or analyzed during this study are available in the CNGB repository under accession number CNP0008414.

## Supplementary Materials

The following supporting information can be downloaded at: https://www.mdpi.com/article/10.3390/plants15020174/s1, Figure S1: GO functional classification of DEGs in aromatic coconuts; Figure S2: Annotation of KEGG pathway of DEGs in aromatic coconut; Figure S3: 2AP peak time and 2AP standard curve; Table S1: Primers used in this study.

## Abbreviations

The following abbreviations are used in this manuscript:

2AP	2-acetyl-1-pyrroline
P5CS	pyrroline-5-carboxylate synthetase
GABA	γ-aminobutyric acid
P5C	pyrroline-5-carboxylic acid
OAT	ornithine aminotransferase
PRODH	proline dehydrogenase
P5CR	pyrroline-5-carboxylate reductase
P5CDH	pyrroline-5-carboxylate dehydrogenase
ROS	reactive oxygen species
POD	peroxidase
SOD	superoxide dismutase
CAT	catalase
SODCP	superoxide dismutase
ABA	abscisic acid
DEGs	differentially expressed genes
GO	Gene Ontology
