# Supplementary material for: Salt Stress Enhances Aroma Component 2-Acetyl-1-pyrroline in Aromatic Coconut (Cocos nucifera Linn.)
Source: Plants (Basel). 2026 Jan 6;15(2):174. doi: 10.3390/plants15020174 (PMC12845143; doi:10.3390/plants15020174)
Supplement: Supplementary file 1 [file plants-15-00174-s001.zip › Figure_S1 .pdf]

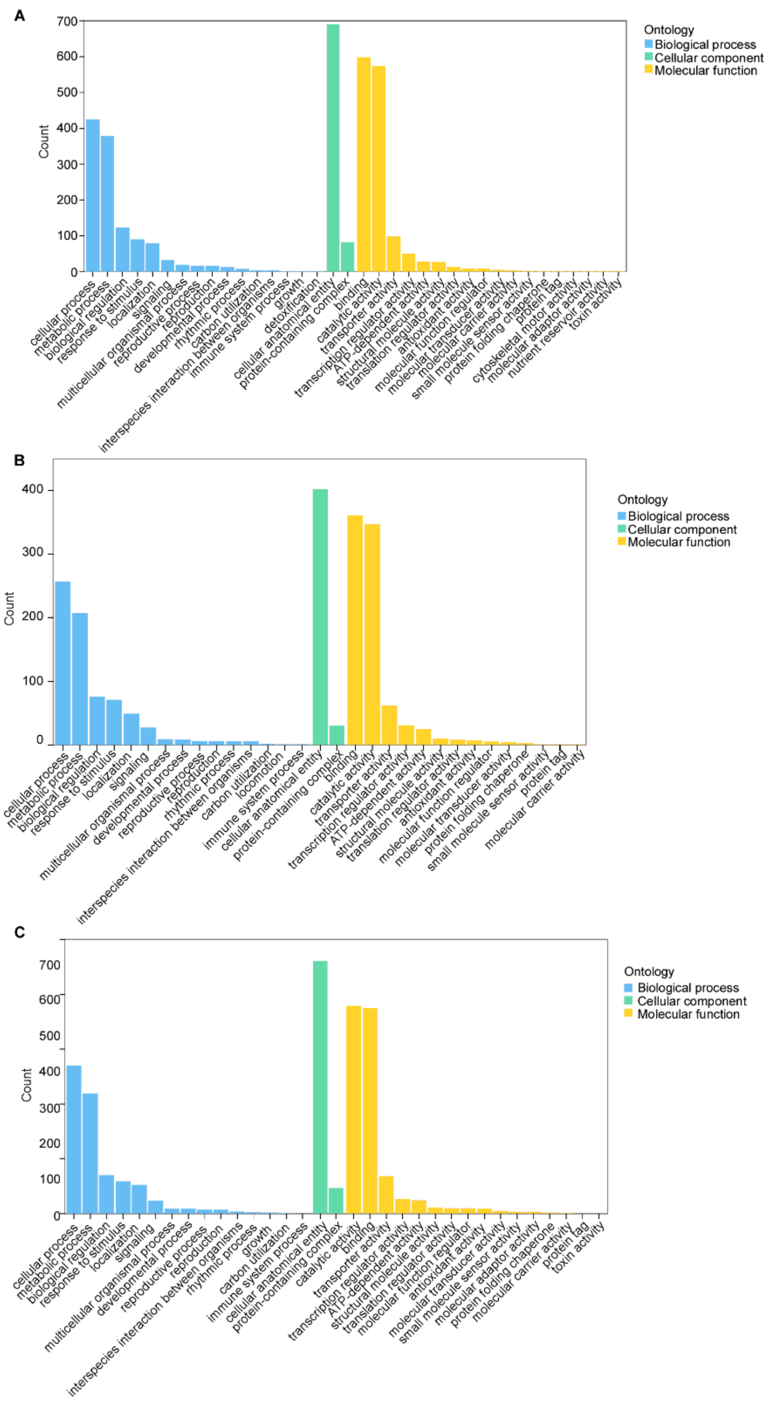

Figure S1 GO functional classification of DEGs in aromatic coconuts. The DEGs obtained by comparing with the control group and the groups treated with different concentrations of NaCl. (A) 0 vs 100 mM NaCl treatments, (B) 0 vs 200 mM NaCl treatments, and (C) 0 vs 300 mM NaCl treatments.
