# Supplementary material for: Salt Stress Enhances Aroma Component 2-Acetyl-1-pyrroline in Aromatic Coconut (Cocos nucifera Linn.)
Source: Plants (Basel). 2026 Jan 6;15(2):174. doi: 10.3390/plants15020174 (PMC12845143; doi:10.3390/plants15020174)
Supplement: Supplementary file 1 [file plants-15-00174-s001.zip › Figure_S2.pdf]

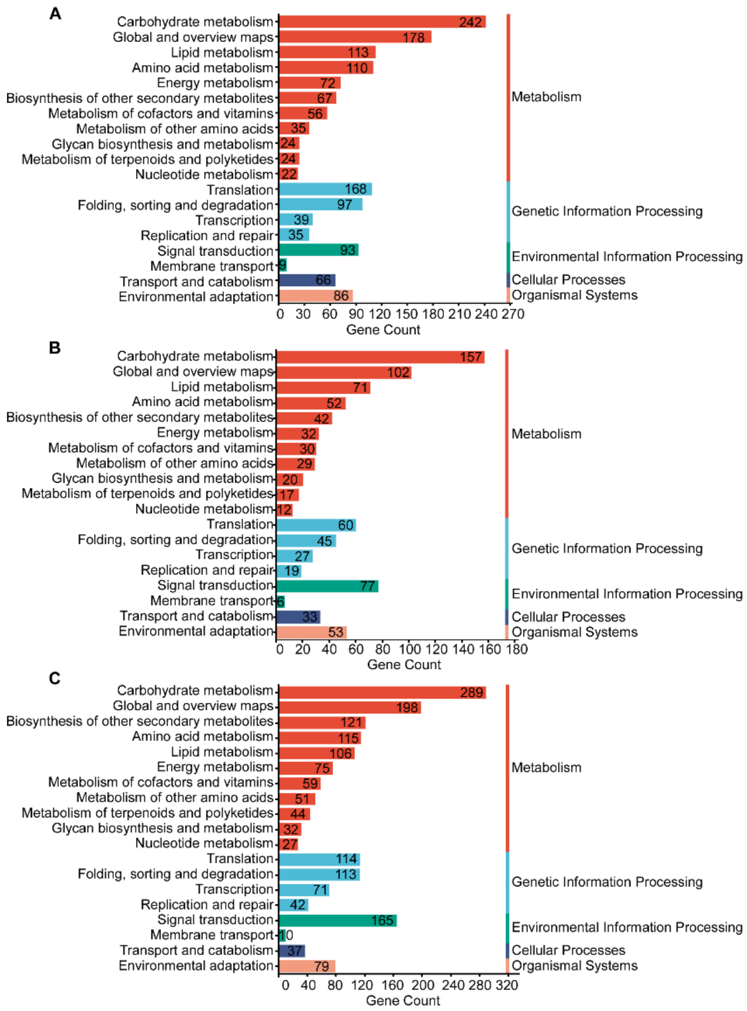

Figure S2 Annotation of KEGG pathway of DEGs in aromatic coconut. The DEGs were obtained by comparing the control group with the groups treated with (A) 100 mM, (B) 200 mM, and (C) 300 mM NaCl.
