# Supplementary material for: Salt Stress Enhances Aroma Component 2-Acetyl-1-pyrroline in Aromatic Coconut (Cocos nucifera Linn.)
Source: Plants (Basel). 2026 Jan 6;15(2):174. doi: 10.3390/plants15020174 (PMC12845143; doi:10.3390/plants15020174)
Supplement: Supplementary file 1 [file plants-15-00174-s001.zip › Figure_S3.pdf]

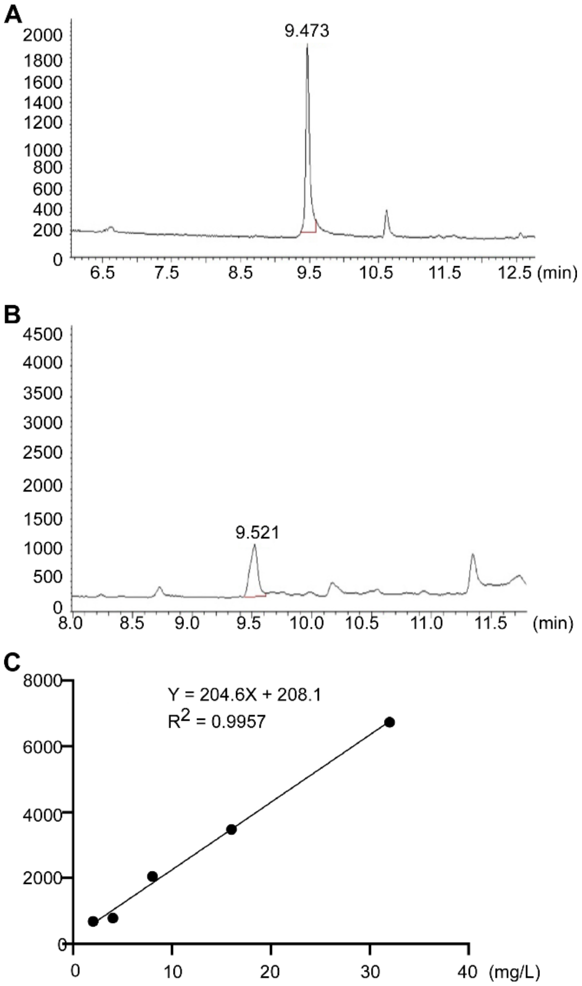

Figure S3 2AP peak time and 2AP standard curve. (A) The peak time of standard 2AP. (B) The peak time of tested samples. (C) The gradient standard is plotted on the standard curve.
