# Supplementary material for: Salt Stress Enhances Aroma Component 2-Acetyl-1-pyrroline in Aromatic Coconut (Cocos nucifera Linn.)
Source: Plants (Basel). 2026 Jan 6;15(2):174. doi: 10.3390/plants15020174 (PMC12845143; doi:10.3390/plants15020174)
Supplement: Supplementary file 1 [file plants-15-00174-s001.zip › Table_S1.pdf]

Supplementary Material

Table S1 Primers used in this study

Primer Name	Primer Sequence (5’-3’)
COCNU_10G005820	F: GAAGATGAAGCAAGGAGAT
	R: CTTTAGGTCTCTGTGGTAT
COCNU_04G008080	F: CTCGTCTTCCAGTCCTTCG
	R: TTGAGGTTTCACAGAGAGA
COCNU_11G007970	F: GACGAAGAGGAGGAGCAGA
	R: CTCTCAATGGGGCATAGGT
	R: TCCCAAGCATTGAATCGTA
COCNU_08G008510	F: TTGGAAAAGGTGGAGCATT
	R: TAAACTCGTCAGCCTCACC
CnACT	F: ATAAAGTATGGCTGATGCTGAGG
	R: CAACAATGCTTGGGAACACA
